# Risk-Reducing Gynecological Surgery in Lynch Syndrome: Results of an International Survey from the Prospective Lynch Syndrome Database

**DOI:** 10.3390/jcm9072290

**Published:** 2020-07-18

**Authors:** Mev Dominguez-Valentin, Toni T. Seppälä, Christoph Engel, Stefan Aretz, Finlay Macrae, Ingrid Winship, Gabriel Capella, Huw Thomas, Eivind Hovig, Maartje Nielsen, Rolf H Sijmons, Lucio Bertario, Bernardo Bonanni, Maria Grazia Tibiletti, Giulia Martina Cavestro, Miriam Mints, Nathan Gluck, Lior Katz, Karl Heinimann, Carlos A. Vaccaro, Kate Green, Fiona Lalloo, James Hill, Wolff Schmiegel, Deepak Vangala, Claudia Perne, Hans-Georg Strauß, Johanna Tecklenburg, Elke Holinski-Feder, Verena Steinke-Lange, Jukka-Pekka Mecklin, John-Paul Plazzer, Marta Pineda, Matilde Navarro, Joan Brunet Vidal, Revital Kariv, Guy Rosner, Tamara Alejandra Piñero, María Laura Gonzalez, Pablo Kalfayan, Julian R. Sampson, Neil A. J. Ryan, D. Gareth Evans, Pål Møller, Emma J. Crosbie

**Affiliations:** 1 Department of Tumor Biology, Institute of Cancer Research, The Norwegian Radium Hospital, Part of Oslo University Hospital, 0379 Oslo, Norway; ehovig@ifi.uio.no (E.H.); moller.pal@gmail.com (P.M.); 2 Department of Abdominal Surgery, Helsinki University Central Hospital, 00029 Helsinki, Finland; toni.seppala@fimnet.fi; 3 Faculty of Medicine, University of Helsinki, 00014 Helsinki, Finland; 4 Institute for Medical Informatics, Statistics and Epidemiology, University of Leipzig, 04107 Leipzig, Germany; christoph.engel@imise.uni-leipzig.de; 5 Institute of Human Genetics, University of Bonn, 53127 Bonn, Germany; Stefan.Aretz@uni-bonn.de (S.A.); claudia.perne@ukbonn.de (C.P.); 6 Center for Hereditary Tumor Syndromes, University Hospital Bonn, 53127 Bonn, Germany; 7 Colorectal Medicine and Genetics, The Royal Melbourne Hospital, 3052 Melbourne, Australia; finlay.macrae@mh.org.au (F.M.); Ingrid.Winship@mh.org.au (I.W.); Johnpaul.plazzer@gmail.com (J.-P.P.); 8 Department of Medicine, University of Melbourne, 3052 Melbourne, Australia; 9 Hereditary Cancer Program, Catalan Institute of Oncology, Insititut d’Investigació Biomèdica de Bellvitge (IDIBELL), ONCOBELL Program, L’Hospitalet de Llobregat, 08908 Barcelona, Spain; capella.gabriel@gmail.com (G.C.); mpineda@iconcologia.net (M.P.); mnavarrogarcia@iconcologia.net (M.N.); Jbrunet@iconcologia.net (J.B.V.); 10 Centro de Investigación Biomédica en Red de Cáncer (CIBERONC), 28029 Madrid, Spain; 11 Department of Surgery and Cancer, St Mark’s Hospital, Imperial College London, London HA1 3UJ, UK; huw.thomas@imperial.ac.uk; 12 Center for Bioinformatics, Department of Informatics, University of Oslo, 0316 Oslo, Norway; 13 Leids Universitair Medisch Centrum, Department of Clinical Genetics, 2300RC Leiden, The Netherlands; m.nielsen@lumc.nl; 14 Department of Genetics, University of Groningen, University Medical Center Groningen, 9713GZ Groningen, The Netherlands; r.h.sijmons@umcg.nl; 15 Scientific Consultant of the Division of Prevention and Genetic Oncology, European Institute of Oncology, Fondazione IRCCS Isrtituto nazionale dei Tumori, 20141 Milan, Italy; lucio.bertario@gmail.com; 16 Division of Prevention and Genetic Oncology, European Institute of Oncology, 20141 Milan, Italy; bernardo.bonanni@ieo.it; 17 Ospedale di Circolo ASST Settelaghi, Centro di Ricerca Tumori Eredo-Familiari, Università dell’Insubria, 21100 Varese, Italy; mariagrazia.tibiletti@asst-settelaghi.it; 18 Gastroenterology and Gastrointestinal Endoscopy Unit, Vita-Salute San Raffaele University, San Raffaele Scientific Institute, 20132 Milan, Italy; cavestro.giuliamartina@hsr.it; 19 Karolinska Institutet, 171 76 Stockholm, Sweden; miriam.mints@ki.se; 20 Department of Gastroenterology, Tel-Aviv Sourasky Medical Center and Sackler Faculty of Medicine, Tel-Aviv University, 64239 Tel Aviv, Israel; nathang@tlvmc.gov.il (N.G.); revitalk@tlvmc.gov.il (R.K.); guyr@tlvmc.gov.il (G.R.); 21 Department of Gastroenterology and Hepatology, Hadassah Medical Center, 91120 Jerusalem, Israel; liorkatz5346@gmail.com; 22 Medical Genetics, Institute for Medical Genetics and Pathology, University Hospital Basel, 4031 Basel, Switzerland; Karl.Heinimann@usb.ch; 23 Hereditary Cancer Program (PROCANHE) Hospital Italiano de Buenos Aires, C1199ABB Ciudad Autónoma de Buenos Aires, Argentina; carlos.vaccaro@hospitalitaliano.org.ar (C.A.V.); tamara.pinero@hospitalitaliano.org.ar (T.A.P.); marialaura.gonzalez@hospitalitaliano.org.ar (M.L.G.); pablo.kalfayan@hospitalitaliano.org.ar (P.K.); 24 University of Manchester & Manchester University NHS Foundation Trust, Manchester M13 9WL, UK; Kate.Green@cmft.nhs.uk (K.G.); fiona.lalloo@cmft.nhs.uk (F.L.); neilryan@nhs.net (N.A.J.R.); gareth.evans@cmft.nhs.uk (D.G.E.); 25 Department of Surgery, Manchester University NHS Foundation Trust and University of Manchester, Manchester M13 9WL, UK; james.hill@mft.nhs.uk; 26 Department of Medicine, Knappschaftskrankenhaus, Ruhr-University Bochum, 44801 Bochum, Germany; Meduni-kkh@ruhr-uni-bochum.de; 27 Department of Medicine, Knappschaftskrankenhaus, Ruhr-University Bochum, 44892 Bochum, Germany; deepak.vangala@rub.de; 28 Department of Gynecology, University Clinics, Martin-Luther University, D-06097 Halle (Saale), Germany; hans.strauss@uk-halle.de; 29 Institute of Human Genetics, Hannover Medical School, 30625 Hannover, Germany; Tecklenburg.Johanna@mh-hannover.de; 30 Medizinische Klinik und Poliklinik IV, Campus Innenstadt, Klinikum der Universität München, 80336 Munich, Germany; elke.holinski-feder@mgz-muenchen.de (E.H.-F.); Verena.Steinke-Lange@mgz-muenchen.de (V.S.-L.); 31 MGZ-Medical Genetics Center, 80335 Munich, Germany; 32 Faculty of Sport and Health Sciences, University of Jyväskylä, 40014 Jyväskylä, Finland; jukka-pekka.mecklin@ksshp.fi; 33 Department of Surgery, Central Finland Health Care District, 40620 Jyväskylä, Finland; 34 Institute of Medical Genetics, Division of Cancer and Genetics, Cardiff University School of Medicine, Cardiff CF14 4XN, UK; sampson@cardiff.ac.uk

**Keywords:** Lynch syndrome, endometrial cancer, ovarian cancer, risk-reducing surgery

## Abstract

Purpose: To survey risk-reducing hysterectomy and bilateral salpingo-oophorectomy (BSO) practice and advice regarding hormone replacement therapy (HRT) in women with Lynch syndrome. Methods: We conducted a survey in 31 contributing centers from the Prospective Lynch Syndrome Database (PLSD), which incorporates 18 countries worldwide. The survey covered local policies for risk-reducing hysterectomy and BSO in Lynch syndrome, the timing when these measures are offered, the involvement of stakeholders and advice regarding HRT. Results: Risk-reducing hysterectomy and BSO are offered to *path*_*MLH1* and *path_MSH2* carriers in 20/21 (95%) contributing centers, to *path_MSH6* carriers in 19/21 (91%) and to *path_PMS2* carriers in 14/21 (67%). Regarding the involvement of stakeholders, there is global agreement (~90%) that risk-reducing surgery should be offered to women, and that this discussion may involve gynecologists, genetic counselors and/or medical geneticists. Prescription of estrogen-only HRT is offered by 15/21 (71%) centers to women of variable age range (35–55 years). Conclusions: Most centers offer risk-reducing gynecological surgery to carriers of *path_MLH1*, *path_MSH2* and *path_MSH6* variants but less so for *path_PMS2* carriers. There is wide variation in how, when and to whom this is offered. The Manchester International Consensus Group developed recommendations to harmonize clinical practice across centers, but there is a clear need for more research.

## 1. Background

Lynch syndrome is one of the most common hereditary cancer syndromes, affecting an estimated 1 in 300 individuals, based on the prevalence of underlying genetic abnormalities in the general population. It is caused by pathogenic variants affecting one of the DNA mismatch repair (MMR) genes (path_MMR): *path_MLH1*, *path_MSH2*, *path_MSH6* and *path_PMS2*, each of which results in different risks for cancer, particularly colorectal, endometrial and ovarian cancer [1].

Women with Lynch syndrome (excepting *path_PMS2*) have a lifetime risk of up to 50% of developing colorectal and endometrial cancer and a lower risk (up to 17%) of developing ovarian cancer [2]. Risk estimates for age-related gynecological cancer in Lynch syndrome vary by gene, as described by the Prospective Lynch Syndrome Database (PLSD). PLSD reported a cumulative incidence of endometrial and ovarian cancer at 75 years of 37% and 11% for *path_MLH1* carriers, 49% and 17% for *path_MHS2* carriers and 41% and 11% for *path_MSH6* carriers, respectively [2]. Notably, female *path_MSH6* carriers are at highest risk of endometrial cancer compared with cancer in other organs [2,3,4].

Whilst colonoscopy surveillance has been associated with improved survival by early detection of colorectal lesions compared with no surveillance [5], there is limited evidence that gynecological cancer surveillance offers a survival benefit due to a lack of high-quality trial data [6]. Nevertheless, a 98% 10-year survival from endometrial cancer has been reported by the PLSD in women who are known path_MMR carriers, suggesting that surveillance and/or increased awareness of the red flag symptoms of gynecological cancers may enable detection at early stages when cure is more likely [4].

In contrast, risk-reducing hysterectomy and BSO have been shown to prevent gynecological cancer in women with Lynch syndrome [7]. The Manchester International Consensus Group recommendations for the management of gynecological cancers in Lynch syndrome suggested that risk-reducing total hysterectomy and BSO should be offered no earlier than 35–40 years of age, following completion of childbearing, to *path_MLH1*, *path_MSH2* and *path_MSH6* carriers. However, the group concluded that there was insufficient evidence to offer risk-reducing surgery to *path_PMS2* carriers, whose risk of gynecological cancers is appreciably lower [6,8]. The prescription of estrogen-only replacement therapy (HRT) until at least natural menopause (~51 years) was strongly recommended for women who undergo risk-reducing surgery [6].

In this report, we describe the current practice for hysterectomy and BSO reported by each PLSD contributing center. We also explore reasons for divergence in the participating centers at the time of the survey.

## 2. Methods

All contributing centers were asked to complete a structured survey of current practice of risk-reducing gynecological surgery by April 2019 (Table 1). Data were collected via a structured questionnaire (with areas for qualitative data collection).

The survey covered questions regarding the current practice for *path_MLH1*, *path_MSH2*, *path_MSH6* and *path_PMS2* carriers, including risk-reducing hysterectomy and BSO and the timing of surgery. We asked each center about the involvement of the patient and healthcare professional stakeholders in initiating discussions about risk-reducing gynecological surgery, whether surgery was actively recommended and if so, for whom.

Data were exported to an excel file, and descriptive statistics were used to catalogue the findings. Local guidelines for gynecological surveillance and the modalities used are shown in Supplementary Table 1.

## 3. Results

### 3.1. Survey Response

We conducted the survey in 31 participating medical centers, from 18 countries. Of the thirty-one centers, 21 (68%) from 12 countries, including Germany (*n* = 5), Finland (*n* = 1), Australia (*n* = 1), Spain (*n* = 1), the United Kingdom (*n* = 2), Norway (*n* = 1), The Netherlands (*n* = 2), Italy (*n* = 3), Sweden (*n* = 1), Israel (*n* = 2), Switzerland (*n* = 1) and Argentina (*n* = 1), completed the survey (Table 2).

### 3.2. Risk-Reducing Hysterectomy and BSO as Measures to Prevent Gynecological Cancer in Path_MMR Carriers

Risk-reducing hysterectomy and BSO are offered in 20/21 (95%) centers for *path_MLH1* and *path_MSH2* carriers. For *path_MSH6* carriers, risk-reducing hysterectomy and BSO are offered in 19/21 (91%) centers. Fourteen out of 21 (67%) centers offer risk-reducing hysterectomy and BSO for *path_PMS2* carriers (Table 2). The most common reported age at which risk-reducing surgery is currently offered is ≥40 years in path_MMR carriers. Prescription of estrogen-only HRT after premenopausal oophorectomy is recommended by 15/21 (71%) centers, across a variable age range (35–55 years).

### 3.3. Involvement of Stakeholders

#### 3.3.1. Risk-Reducing Hysterectomy

Of the 21 centers, 11 provided information about which stakeholders are involved in initiating discussions about risk-reducing hysterectomy with Lynch syndrome carriers. Ten (91%) centers stated that healthcare professionals (mainly gynecologists, genetic counselors and medical geneticists) offer advice about risk-reducing hysterectomy to women with Lynch syndrome, and 6/9 (67%) centers that provided further information stated that risk-reducing hysterectomy is actively recommended. Of the 21 centers, only 4 provided information about patient engagement in discussions about risk-reducing hysterectomy. Of these four centers, one (25%) reported that risk-reducing hysterectomy is only provided upon request by Lynch syndrome carriers.

#### 3.3.2. BSO

With regards to risk-reducing BSO, 10/21 centers provided information about stakeholder involvement in these discussions. Nine (90%) centers stated that healthcare professionals (mainly gynecologists, genetic counselors and medical geneticists) offer advice to Lynch syndrome carriers about risk-reducing BSO. In 9/10 centers, risk-reducing BSO is advocated by healthcare professionals, and in 5/9 (56%), it is actively recommended. Of the 21 centers, 6 provided information about the involvement of patients in discussions about risk-reducing BSO. Three of those centers (50%) stated that surgery is only provided upon request by Lynch syndrome carriers.

## 4. Discussion

Here, we provide insight into the current management of gynecological cancer risk in women with Lynch syndrome across participating PLSD centers. We received data from 21/31 (68%) PLSD centers, incorporating practices from 12 countries worldwide. There was global agreement (>90%) for offering both hysterectomy and BSO to *path_MLH1*, *path_MSH2* and *path_MSH6* carriers after the age of 40 years. The reported age at which risk-reducing surgery is offered is later than the minimum age (35 years) for *path_MLH1* and *path_MSH2* carriers suggested by the recent expert consensus statement [6], which was based on the reported rapidly rising risk of gynecological cancers for *path_MSH2* and *path_MSH6* carriers from that age onwards [2]. Despite the very low risk for gynecological cancer in *path_PMS2* carriers, risk-reducing hysterectomy and BSO are still offered by 67% of the responding centers. Interestingly, we identified no major differences between the countries, but there are some discrepancies between centers from the same country (e.g., the UK, The Netherlands and Israel). Of note, the survey was taken one year ago (April 2019), and some retrospective and prospective data studies on ovarian and endometrial cancer risk in Lynch syndrome were just being published [8,9,10]. This may explain the discrepancies between centers, especially for *path_PMS2* carriers, whereby some centers (e.g., The Netherlands) have adapted their practice, but changes have not been incorporated into national guidelines yet, whilst other centers (e.g., Switzerland) rely on recommendations from European and US guidelines/statements.

Decisions about risk-reducing surgery reflect discussions between women and healthcare providers that incorporate a range of issues, including personalized risk calculations, advantages/disadvantages of risk-reducing surgery, gynecological health, medical co-morbidity, family planning, and women’s values and preferences. The complexity of these individualized discussions cannot be captured in a simple survey of this nature. Nevertheless, we sought to understand more about the drivers for these discussions, whether healthcare professionals are active or passive in providing risk-reducing gynecological surgery, and whether this differs by center, country, surgery type (hysterectomy versus BSO) and affected gene. Most centers offer risk-reducing hysterectomy and/or BSO, but there is a huge variation between centers as to whether surgery is actively recommended or provided only upon patient request. This likely reflects inconsistency in national and international guidelines underpinned by a lack of high-quality published studies. There is a clear unmet clinical need for more research in this area to guide a consistent approach to risk-reducing gynecological surgery for Lynch syndrome carriers irrespective of where an individual woman lives and who is involved in her care [11].

The prescription of HRT following premenopausal oophorectomy is recommended in 71% of the centers from 35 years for a variable duration of up to 55 years of age. The reported ages at which HRT is offered is not in line with the strong recommendation by the Manchester International Consensus Group that suggests HRT is offered until at least the age of natural menopause (~51 years) [6]. Some centers do not prescribe HRT at all; it is notable that the current National Comprehensive Cancer Network (NCCN) guidelines for risk management for women with Lynch syndrome do not mention prescription of HRT [12]. However, estrogen protects against colorectal cancer [13,14,15,16,17,18], which is particularly relevant for Lynch syndrome carriers, as well as protecting bone and cardiovascular health [19,20]. Many of the well-publicized harms associated with HRT relate to its prolonged use in older women and specifically from the progestin component that is not required when the uterus is removed [21,22].

Risk-reducing hysterectomy and/or BSO has been reported to prevent cancer and/or to improve survival in women with Lynch syndrome [7]. For women who choose not to undergo risk-reducing surgery, an understanding of the ‘red flag’ symptoms for these cancers is important to trigger prompt referral for urgent investigation [23]. A woman’s personal risk should be used to provide individualized counselling regarding the need for risk-reducing surgery and the optimal timing of this.

## 5. Conclusions

This study provides a snapshot of the preventive gynecological recommendations provided to women with Lynch syndrome by 21 centers from 18 countries worldwide. There is a wide variation in how, when and to whom risk-reducing gynecological surgery is offered. The Manchester International Consensus Group developed guidance to harmonize the care offered to women with Lynch syndrome but noted the lack of high-quality research in this area. There is a clear need for further research so that women with Lynch syndrome can expect and receive consistent, evidence-based care for the management of their gynecological cancer risk.

## Figures and Tables

**Table 1:** Survey covering questions regarding the current practice with respect to prophylactic hysterectomy and/or bilateral salpingo-oophorectomy (BSO) in *path_MMR* carriers.

Current Practice		*Path_MLH1* Carrier	*Path_MSH2* Carrier	*Path_MSH6* Carrier	*Path_PMS2* Carrier
Prophylactic hysterectomy	Available (Y/N)				
	If so, at which age				
	If so, only if patient asks *				
	If so, to be mentioned by counsellor *				
	If so, to be advocated by counsellor *				
Prophylactic oophorectomy	Available(Y/N)				
	If so, at which age				
	If so, only if patient asks **				
	If so, to be mentioned by counsellor **				
	If so, to be advocated by counsellor **				
	If premenopausal oophorectomy, HRT to which age?				

*/** mutually exclusive, answer only one for each gene; */** please specify who the counsellor would be (more than one if applicable), e.g. gynecologist, medical geneticist, genetic counsellor, GP, other.

**Table 2:** Currently recommended practice for females with path_MMR variants in the Prospective Lynch Syndrome Database (PLSD) by country.

Countries	Number of Centers	*Path_MLH1 and Path_MSH2 Carriers*		*Path_MSH6 Carriers*		*Path_PMS2 Carriers*	
		Prophylactic Hysterectomy	Prophylactic BSO	Prophylactic Hysterectomy	Prophylactic BSO	Prophylactic Hysterectomy	Prophylactic BSO
Germany	5	5	5	5	5	5	5
Finland	1	1	1	1	1	1	1
Australia	1	1	1	1	1	1	1
Spain	1	1	1	1	1	1	1
UK	2	2	2	2	2	1	1
Norway	1	1	1	1	1	1	1
The Netherlands	2	2	2	2	2	0	0
Italy	3	3	3	3	3	1	1
Sweden	1	1	1	1	1	1	1
Israel	2	2	2	1	1	1	1
Switzerland *	1	0	0	0	0	0	0
Argentina	1	1	1	1	1	1	1
*Total*	21	20	20	19	19	14	14

*: There is no national guidelines or statements for female Lynch syndrome patients yet; BSO: bilateral salpingo-oophorectomy.
